# Comparison of Toxicities to *Vibrio fischeri* and Fish Based on Discrimination of Excess Toxicity from Baseline Level

**DOI:** 10.1371/journal.pone.0150028

**Published:** 2016-02-22

**Authors:** Xiao H. Wang, Yang Yu, Tao Huang, Wei C. Qin, Li M. Su, Yuan H. Zhao

**Affiliations:** State Environmental Protection Key Laboratory of Wetland Ecology and Vegetation Restoration, School of Environment, Northeast Normal University, Changchun, Jilin, P. R. China; Northwest Fisheries Science Center, NOAA Fisheries, UNITED STATES

## Abstract

Investigations on the relationship of toxicities between species play an important role in the understanding of toxic mechanisms to environmental organisms. In this paper, the toxicity data of 949 chemicals to fish and 1470 chemicals to *V*. *fischeri* were used to investigate the modes of action (MOAs) between species. The results show that although there is a positive interspecies correlation, the relationship is poor. Analysis on the excess toxicity calculated from toxic ratios (TR) shows that many chemicals have close toxicities and share the same MOAs between the two species. Linear relationships between the toxicities and octanol/water partition coefficient (log K_OW_) for baseline and less inert compounds indicate that the internal critical concentrations (CBRs) approach a constant both to fish and *V*. *fischeri* for neutral hydrophobic compounds. These compounds share the same toxic mechanisms and bio-uptake processes between species. On the other hand, some hydrophilic compounds exhibit different toxic effects with greatly different log TR values between *V*. *fischeri* and fish species. These hydrophilic compounds were identified as reactive MOAs to *V*. *fischeri*, but not to fish. The interspecies correlation is improved by adding a hydrophobic descriptor into the correlation equation. This indicates that the differences in the toxic ratios between fish and *V*. *fischeri* for these hydrophilic compounds can be partly attributed to the differences of bioconcentration between the two species, rather than the differences of reactivity with the target macromolecules. These hydrophilic compounds may more easily pass through the cell membrane of *V*. *fischeri* than the gill and skin of fish, react with the target macromolecules and exhibit excess toxicity. The compounds with log K_OW_ > 7 exhibiting very low toxicity (log TR < –1) to both species indicate that the bioconcentration potential of a chemical plays a very important role in the identification of excess toxicity and MOAs.

## Introduction

Information regarding aquatic toxicity is required in the assessment of the toxicity of organic chemicals to marine and freshwater organisms. Discrimination of excess toxicity from narcotic level plays an important role in the study of modes of action (MOAs) for organic chemicals [1, 2]. Until now, many studies have been performed on the identification of MOAs for different kinds of organic chemicals [3, 4, 5]. Chemicals are generally categorized into baseline chemicals (non-polar narcosis), less-inert chemicals (polar narcosis), reactive chemicals (weak acid respiratory uncoupling, free-radical formation, as well as electrophilic reactions) and specifically acting chemicals [6].

The narcotic chemicals (baseline and less inert chemicals) have toxic effects through the disruption of the proper function of the cell membrane and can be quantified by using the hydrophobic parameter log K_OW_ (octanol/water partition coefficient). Linear relationships have been observed between log K_OW_ and the toxicities of these narcotics to a variety of species [7, 8, 9, 10]. Reactive chemicals exhibit significantly greater toxicity than that predicted from hydrophobicity alone due to the existence of a more specific interaction with organisms. For the identification of reactive compounds, the toxic ratio (TR) was employed to discriminate the excess toxicity from narcotic effect. The excess toxicity expressed as toxic ratio (TR) is calculated from the predicted baseline toxicity divided by the experimental values [6]. The threshold of log TR = 1 which is based on the distribution of fish toxicity data is generally used to discriminate the excess toxicity from narcotic effect.

Investigations on the interspecies correlation showed that although there is a positive interspecies correlation between species, significant differences in excess toxicity have been observed for some compounds between difference species. This suggests that some compounds may share the same MOAs between species, but some may not [11, 12]. However, toxic mechanisms are not only dependent on the structural characteristics of chemicals, but also on the physiological characteristics of test organisms, species sensitivity, bioconcentration potential, exposed time and even the environmental conditions surrounding the aquatic organisms. Comparison of the toxicities between species is not only useful in the hazardous and risk assessment of chemicals to organisms in the environment, but also helpful for the understanding of toxic mechanisms. Fish and bacteria belong to different trophic levels in the ecosystem. They are commonly used as test organisms for the investigation of toxicology for environmental toxicants. Bioluminescence inhibition of *Vibrio fischeri* (*V*. *fischeri*) and 50% lethal concentration of fish have been widely used as a quick and convenient bioassay for the evaluation of toxic effects caused by organic pollutants in the environment [8, 13, 14, 15]. A significant correlation has been observed between *V*. *fischeri* and fish toxicities [12].

In this paper, toxicity data of 2043 compounds (1470 to *V*. *fischeri* and 949 to fish) compiled from literature and databases were used to compare the toxicities between fish and *V*. *fischeri*. The compounds were classified into different classes or homologues based on the substituted functional groups and MOAs of the compounds. The toxic ratios (TR) were calculated for the classified compounds. The aims of the present work are: First, to investigate interspecies correlation between the toxicity data of overlapping compounds to fish and *V*. *fischeri*; Second, to develop baseline and less inert models and use them to discriminate the excess toxicity from narcotic level for classified compounds; Third, to compare the toxicities between fish and *V*. *fischeri* for different classes of compounds and to investigate the species-based MOAs for hydrophobic and hydrophilic compounds.

## Materials and Methods

### Toxicity data to *V*. *fischeri* and fish

*V*. *fischeri* toxicity data of 50% inhibition of bioluminescence after 5, 15 or 30 min exposure expressed as IBC_50_ (mol/L) for 1470 compounds were taken from several references [9, 10, 13, 15–20]. Analysis shows that there is no significant difference between these three toxicity endpoints. Where possible, 15 min IBC_50_ values were used in this paper. If 15 min endpoints are not available, 30 or 5 min endpoints were used instead. All the IBC_50_ values were converted to the logarithm of the IBC_50_ in mol/L (i.e. log 1/IBC_50_). The compounds were classified into different classes/homologues based on the structures and substituted functional groups. The log 1/IBC_50_ collected from different references for different endpoints, together with names, SMILES and CAS numbers can be found in Table A in S1 File.

Fish toxicity data of 50% lethal rate within 96 h expressed by LC_50_ (mol/L), for 949 compounds were taken from several references and a database. The toxicities to guppy (*Poecilia reticulata*) and rainbow trout (*Oncorhynchus mykiss*) were obtained from two references [21, 22]. The toxicities to fathead minnow (*Pimephales promelas*) were compiled from different published papers (the references in details see Table B in S1 File). The toxicities to medaka (*Oryzias latipes*) were extracted from a CHRIP (Chemical Risk Information Platform) database (http://www.safe.nite.go.jp/english/db.html). Significant correlations were observed between the log 1/LC_50_ values for four fish species. Therefore, a single combined toxicity data set for fish was constructed in this paper [11]. The log 1/LC_50_ collected from different references, together with names, SMILES and CAS numbers can be found in Table B in S1 File.

The total number of compounds reported in this paper is 2043 compounds. The toxicity values to both species can be found in Table C in S1 File.

### Excess toxicity

The toxic ratio (TR), the predicted baseline or minimum toxicity (T _pred_) over the experimentally determined value (T _exp_), was used to evaluate and discriminate the excess toxicity from baseline level [1, 6, 23, 24].

$$TR=T_{\;pred\;(baseline)}/T_{\;\exp}$$ (1)

$$\log\;TR=\log\;1/T_{\;\exp}\;\text{-}\;\log\;1/T_{\;pred\;(baseline)}=\text{Residual}$$ (2)

Where, T is the toxicity value to fish or *V*. *fischeri* (i.e. LC_50_ or IBC_50_). A threshold of log TR = 1 was used to discriminate excess toxicity from baseline level (non-polar narcotic effect). A log TR-value from minus one to one is assumed as baseline or less inert toxicity. A log TR-value significantly greater than one is assumed as excess toxicity due to the existence of a reactive or more specific MOA.

### Molecular descriptors and statistical analysis

The octanol/water partition coefficients (K_OW_) were obtained from the EPISuite programme (version 4.0, http://www.epa.gov/oppt/exposure/pubs/episuitedl.htm). Where possible, measured log K_OW_ values were used in preference to calculated values. The linear regression analysis was performed using a least-squares linear regression with the Minitab software (version 14). The following descriptive information is provided for each regression: number of observations used in the analysis (N), coefficient of determination (R^2^), standard error of the estimate (S) and Fisher’s criterion (F).

## Results

### Interspecies correlation between toxicities of fish and *V*. *fischeri*

**Fig 1:**
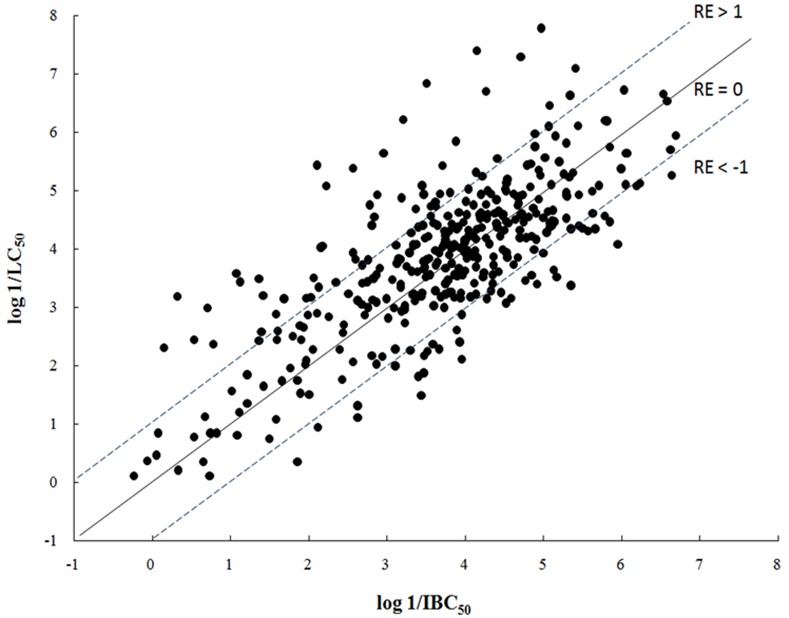
Interspecies correlation of toxicities between fish and *V*. *fischeri*. RE (residual) = log 1/LC_50_ –log 1/IBC_50._

Fig 1 is a plot of log 1/LC_50_ of fish against log 1/IBC_50_ of *V*. *fischeri* for 376 overlapping chemicals. The Pearson correlation coefficient (R) for the 376 chemicals is 0.72 and a very significant outlier (acetonitrile) has been observed between the toxicities to fish and *V*. *fischeri*, with toxicity values of 4.60 and –1.11, respectively. Exclusion of the outlier does not greatly improve the correlation. The interspecies regression equation is:

$$\log\;1/LC_{50}=0.729\;\log\;1/IBC_{50}+1.15$$ (3)

N = 375 S = 0.89 R^2^ = 0.55 F = 449

Although log 1/LC_50_ of fish is positively correlated to log 1/IBC_50_ of *V*. *fischeri*, the relationship is poor with R^2^ = 0.55. Considerable scatter in the interspecies correlation is observed in the interspecies correlation (see Fig 1). The equation slope less than one and intercept higher than zero suggests that there is a difference in species sensitivity. Fig 1 shows the dividing line of species sensitivity (residual = 0), together with the lines of residual = 1, residual = –1 between the toxicities of fish and *V*. *fischeri* (residual = log 1/LC_50_ –log 1/IBC_50_). This figure indicates that fish are slightly more sensitive than *V*. *fischeri* with the average residual of 0.16. Some compounds are more toxic to fish (residual > 1) and some are more toxic to *V*. *fischeri* (residual < –1). This indicates that there is chemical-specific species sensitivity between fish and *V*. *fischeri*. Inspection of the characteristics of compounds suggests that fish are more sensitive to hydrophobic compounds and *V*. *fischeri* are more sensitive to hydrophilic compounds in toxicity. For examples, eight out of twenty one alkanes exhibit greater toxicity (residual > 1) to fish than that to *V*. *fischeri* with the average residual of 0.72. Seven of sixteen amines exhibit greater toxicity (residual < –1) to *V*. *fischeri* than that to fish with the average residual of –0.16 (see Table C in S1 File). The above analysis suggests that some chemicals may share the same toxic modes of action between the two species, but some may not. Because of the limited number of toxicity data for the overlapping compounds to both species, it is impossible to investigate the species sensitivity and the toxic mechanisms of action for all homologues/classes. It is noteworthy that above comparison is only based on the acute toxicity data for 15 min exposure to *V*. *fischeri* and 96 h exposure to fish, not the sub-acute or chronic toxicity data. Some compounds can have different toxic effects to same species at sub-acute or chronic exposure. Many factors, such as exposure duration, bioconcentration potential, ionization and experimental error, can affect the difference of toxicities between *V*. *fischeri* and fish species. This will be discussed below.

### Development of models for baseline and less inert compounds

The interspecies correlation is a useful method to compare the similarities and differences in toxicities between species. But it cannot be used to investigate whether or not the compounds share the same MOAs between species [12]. To investigate whether the compounds share the same or different MOAs, the toxic ratio (TR) was employed in this paper. According to Eq 1 or Eq 2, baseline models need to be developed to calculate the baseline toxicities and the TR values for the studied compounds. The compounds we used to develop the baseline models for the two species are substituted aliphatic and aromatic compounds, such as alkanes, alcohols, ethers, ketones, alkyl benzenes and their chlorinated derivatives. They are widely categorized as baseline (or called non-polar narcotic) compounds in literature [6,25]. The linear regression analysis between log K_OW_ and the toxicities for baseline compounds to fish (log 1/LC_50_) and *V*. *fischeri* (log 1/IBC_50_), respectively, are:

$$\log\;1/LC_{50}=0.883\;\log\;K_{OW}+1.16$$ (4)

N = 121 S = 0.32 R^2^ = 0.94 F = 1929

$$\log\;1/IBC_{50}=0.994\;\log\;K_{OW}+0.863$$ (5)

N = 97 S = 0.50 R^2^ = 0.89 F = 748

At the same time, linear regression analysis between log K_OW_ and the toxicities for less inert compounds (e.g. substituted anilines and phenols), were also carried out to fish and *V*. *fischeri*, respectively (Eqs 6 and 7). These two equations were used to discriminate less inert compounds from baseline level for fish and *V*. *fischeri* toxicities, respectively (see discussion section below).

$$\log\;1/LC_{50}=0.638\;\log\;K_{OW}+2.50$$ (6)

N = 86 S = 0.34 R^2^ = 0.84 F = 455

$$\log\;1/IBC_{50}=0.708\;\log\;K_{OW}+2.26$$ (7)

N = 76 S = 0.34 R^2^ = 0.79 F = 279

**Fig 2:**
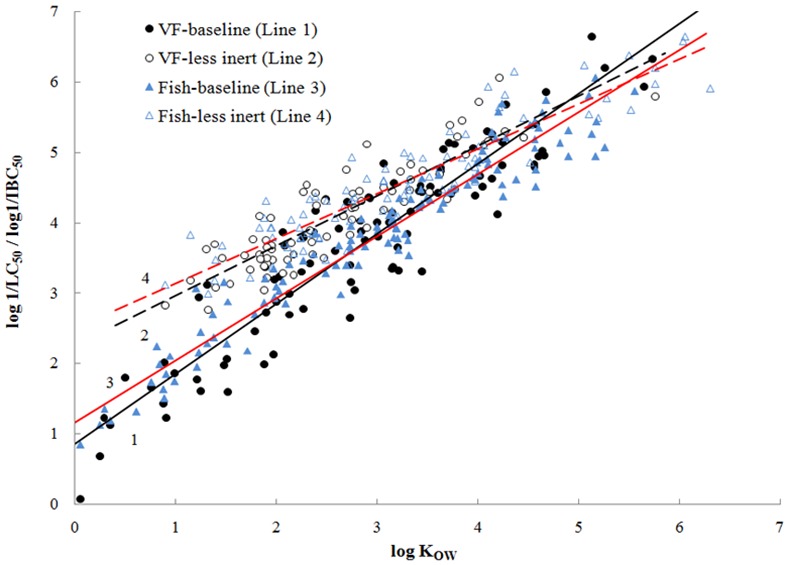
Relationships between log K_OW_ and toxicities to fish and *V*. *fischeri* (VF) for baseline and less inert compounds.

Fig 2 shows the plots of the toxicities against log K_OW_ and their fitting lines for baseline and less inert compounds to both species. It should be noted that the compounds with log K_OW_ > 7 and log TR > 1 or < –1 were removed in the baseline model development. The reason for removing these compounds will be discussed below. Eqs 4 or 5 are used to predict the minimum toxicities and Eqs 6 or 7 are used to calculate the less inert toxicities to fish or *V*. *fischeri* for all the studied compounds.

### Discrimination of excess toxicity to fish and *V*. *fischeri*

The log TR values used to evaluate the excess toxicity of fish and *V*. *fischeri*, as calculated from Eq 2 and Eq 4 or Eq 5, respectively, for all the compounds are listed in Table C in S1 File. The summary statistics for classified compounds with log TR < –1 (outlier), –1 ≤ log TR ≤ 1 (baseline and less inert toxicity), log TR > 1 (excess toxicity) for fish and *V*. *fischeri* toxicities, respectively, are listed in Table 1. The toxic ratios for the classes with only one or two compounds are not listed in Table 1. They are listed in Table C in S1 File. The N_B_/N_L_ listed in Table 1 are numbers of compounds predicted as baseline over that predicted as less inert compounds. For a compound, if the absolute residual predicted from baseline model (Eqs 4 or 6) is less than that predicted from less inert model (Eqs 5 or 7), it will be classified as a baseline compound. Otherwise, it will be classified as a less inert compound.

**Table 1:** The number of classified chemicals in each class (N) and the number of chemicals predicted as outliers (log TR < -1), the ratios of compounds predicted as baseline or less inert toxicity (N_B_/N_L_) and the number of compounds with excess toxicity (log TR > 1) to *V*. *fischeri* and fish, respectively

	Classes	MOA (Ref.)	*V*. *fischeri*				Fish			
			N logTR<-1 N_B_/N_L_ logTR>1				N logTR<-1 N_B_/N_L_ logTR>1			
1	Compounds used in baseline model (Alkanes, cycloalkanes alcohols, ethers, ketones, benzenes with alkyl, fluoro or chloro groups)	B1	116	11	97/0	8	122	0	121/0	1
2	Compounds used in less inert model (Phenols and anilines with alkyl, fluoro or chloro groups)	B1	85	2	1/63	19	86	0	0/27	14
3	Alkanes with bromo group	B1	5	1	4/0	0	9	0	7/1	1
4	Alkenes, dienes, alkynes with chloro group	B1	9	1	6/2	0	18	0	12/5	1
5	Allylic and propargyl halogens	R1,3	7	0	2/3	2	6	0	0/1	5
6	β-Halogenated alcohols	R2	8	0	4/3	1	8	0	4/1	3
7	Diols	B1	7	0	3/1	3	4	1	3/0	0
8	α,β-Unsaturated alcohols	R1,3	0	0	0/0	0	20	1	7/2	10
9	Alchohol-ethers		5	0	2/2	1	9	0	9/0	0
10	Aldehydes	R1	9	0	2/3	4	8	0	0/1	7
11	α,β-Unsaturated aldehydes	R1	8	0	0/2	6	2	0	0/0	2
12	α-Halogenated ketones	R3	5	0	0/1	4	0	0	0/0	0
13	Diones	R2,3	11	0	1/1	9	3	1	1/0	1
14	α,β-Unsaturated ketones	R1	2	0	0/0	2	2	0	2/0	0
15	Esters, bromo esters and diesters		18	0	11/7	0	13	0	3/7	3
16	α-Halogenated esters	R2,3	7	0	0/2	5	1	0	1/0	0
17	α,β-Unsaturated esters	R1	3	0	2/1	0	22	0	3/3	16
18	Carboxylic acids with fluoro or chloro group*		36	14	15/2	5	7	0	5/1	1
19	Diacids*		4	2	2/0	0	2	0	0/0	2
20	α,β-Unsaturated carboxylic acids*	R1	2	0	0/0	2	3	0	0/1	2
21	Primary mono amines*	L1,R3	9	1	4/1	3	21	0	8/12	1
22	Secondary mono amines*	B1	2	0	1/0	1	3	0	2/1	0
23	Tertiary amines*	B1	1	0	1/0	0	5	1	2/1	1
24	Diamines and poly amines*	R3	3	0	0/0	3	8	0	1/1	6
25	Nitriates, chloro nitriates and cyclo nitriates		11	0	1/4	6	1	0	0/0	1
26	Alkyl nitriles, chloro and bromo nitriles, dinitriles and α,β-unsaturated nitriles		20	1	6/2	11	16	0	1/5	10
27	Alkyl hydrazines*	R1	2	0	0/0	2	2	0	0/0	2
28	Amides, α-chloro amides, α,β-unsaturated amides	R1-3	6	0	1/1	4	8	0	4/0	4
29	Ureas		5	1	0/1	3	0	0	0/0	0
30	Epoxides	R1,3	2	0	1/0	1	4	0	1/0	3
31	Thiols, thioethers, dithioethers and disulphides	R1-4	7	0	3/2	2	20	0	10/4	6
32	Thioureas		10	0	0/1	9	0	0	0/0	0
33	Thiophosphates	R1-3	11	0	3/1	7	5	0	3/0	2
34	Phosphates and phosphonic acids*	R2	5	0	1/0	4	4	1	2/1	0
35	Bromo or indo benzenes		12	0	6/6	0	3	0	0/3	0
36	Benzyl chlorides and bromides	R2,3	11	0	2/3	6	5	0	1/1	3
37	Phenyl ethenes or acetylenes	R2,3	5	0	1/3	1	5	0	2/1	2
38	Phenyl alcohols		15	0	1/1	13	8	1	6/1	0
39	Alkoxy benzenes		10	0	1/6	3	5	0	5/0	0
40	Benzaldehydes with alkyl, halogen or alkoxy group	R1	18	0	4/7	7	16	0	2/6	8
41	Benzaldehydes with alkyl, hydroxy, amino, nitro and cyano groups and benzdialdehydes	R1	13	0	0/3	10	16	0	2/4	10
42	Phenones with halogen, alkoxy, ketone group and diphenones		21	0	2/5	14	7	0	1/2	4
43	Benzoates and phenylalkyl esters		13	2	0/2	9	4	0	1/1	2
44	Phthalates		5	2	1/1	1	7	1	2/3	1
45	Benzoic acids with alkyl, halogen, ketone, ester, hydroxy, nitro, amino or cyano group*		30	0	4/10	16	7	2	4/1	0
46	Phenylalkyl acids with alkyl, halogen, alkoxy, hydroxy, nitro or amino groups and α,β-unsaturated acids*		36	1	16/7	12	0	0	0/0	0
47	Benzoyl chlorides	R1	9	0	0/0	9	0	0	0/0	0
48	Phenols with bromo group*		7	0	3/1	3	4	0	2/2	0
49	Phenols with alcohol, alkoxy, ketone or ester group		29	1	4/9	15	7	0	2/5	0
50	Nitro phenols*	L1	11	0	0/5	6	5	0	1/2	2
51	Cyano phenols		3	0	0/0	3	3	0	2/1	0
52	Hydroxyquinones	R3	12	0	1/1	10	7	0	0/2	5
53	Nitro anilines with halogen or alkoxy group	L1	18	0	0/6	12	7	0	0/6	1
54	aniline-NH		5	0	0/0	5	5	0	3/2	0
55	aniline-N		7	0	1/2	4	5	0	4/1	0
56	Benzenediamines	R3	8	0	0/0	8	5	0	0/0	5
57	Aminophenols*	R3	7	1	1/0	5	5	0	0/2	3
58	Phenylalkyl amines with halogen or alkoxy group*		10	0	1/2	7	5	0	0/4	1
59	Mono nitrobenzenes with alkyl or halogens	L1	41	0	13/18	10	19	0	3/14	2
60	Mono nitrobenzenes with alcohol, alkoxy, ketone, ester, or cyano group		25	0	0/4	21	7	0	1/2	4
61	Dinitrobenzenes and trinitrobenzenes with halogen, alkoxy, hydroxy, alcohol, amino or cyano group	R2,3	30	0	1/1	28	14	0	0/3	11
62	Benzo nitriles with alkyl, halogen alkoxy, ketone or ester groups and benzo dinitriles		19	0	1/5	13	8	0	3/2	3
63	Phenylalkyl nitriles		14	0	0/0	14	0	0	0/0	0
64	Benzenes with isocyano group		11	0	3/7	1	0	0	0/0	0
65	Phenyl hydrazines	R1	3	0	0/0	3	2	0	0/0	2
66	Benzamides with halogen, alkoxy, hydroxy, amino or nitro group		8	0	0/1	7	10	0	2/5	3
67	Anilides with halogen, alkoxy or ether group		8	1	3/3	1	6	0	1/4	1
68	Phenyl ureas with halogen or alkoxy groups		11	1	6/3	1	1	0	0/1	0
69	Thiophenols with alkyl, halogen or alkoxy groups*	R4	28	0	7/11	10	1	0	0/0	1
70	Benzenes with thiol, thiocyano, thiocyanate, thionitrile, thioamide, thiourea, thiosemicarbazide, sulphonamide, thiocarbamate, sulfonyl, sulfone or sulfonic acid group	R1	36	1	0/5	30	5	0	3/0	2
71	Pyridines with alkyl or halogen group	L1	19	0	6/5	8	11	0	6/1	4
72	Pyridines with alcohol, alkoxy, aldehyde, ketone, ester or carboxylic acid group		16	0	1/2	13	5	0	0/1	4
73	Hydroxy pyridines		5	0	2/2	1	5	0	4/1	0
74	Amino and diamino pyridines*		10	0	0/1	9	5	0	1/1	3
75	Pyridines with nitro or cyano group		7	0	0/1	6	2	0	1/0	1
76	Pyrimidines		4	0	0/1	3	2	0	0/1	1
77	Triazoles, tetrazoles or thiazoles		7	0	0/2	5	0	0	0/0	0
78	Biphenyls with alkyl and chloro group		5	0	3/1	1	2	0	2/0	0
79	Biphenyls with hydroxy or amino group		8	0	0/4	4	6	0	0/5	1
80	Diphenyl alkanes, alcohols, ethers, ketones, esters, amines or amides		16	2	66	2	14	0	4/10	0
81	Diphenyl alkanes, ethers, ketones, esters or amines with hydroxy or amino group		19	2	2/6	9	20	2	2/12	4
82	Phenyl pyridyl compounds		8	0	2/1	5	3	0	1/2	0
83	Naphthalenes		5	0	1/4	0	8	0	5/3	0
84	Naphthalenes with hydroxy or amino group		8	0	0/2	6	5	0	0/1	4
85	PAHs	R3	25	13	7/3	2	4	0	2/1	1
86	Quinolines		32	0	7/13	12	3	0	1/1	1
87	Benzothiazoles		3	0	0/0	3	5	0	0/3	2

MOA (ref.): Mode of action identified by literature and references (B: identified as baseline compounds (or non-polar narcotics); L: identified as less inert compounds (or polar narcotics); R: identified as reactive or specific reactive compounds; Ref. 1: Verhaar et al., 1992; Ref. 2: Russom et al., 1997; Ref. 3: Enoch et al., 2011; Ref. 4: Schwöbel et al., 2011).

*: classes with ionizable compounds.

The similarities and differences in toxic effects of the classified compounds to fish and *V*. *fischeri* can be seen clearly from the summary statistics. Table 1 shows that most classes can be identified as the same MOAs between the two species. The alkanes, alkenes, alcohols, ketones, ethers and benzenes with alkyl and halogens show baseline toxicity to both species. Phenols and anilines exhibit less inert toxicity to both species, although phenols are slightly more toxic to *V*. *fischeri*, whereas anilines are more toxic to fish (class 2). Aldehydes (class 11), diamines (class 24), nitriles (class 26), hydroxy benzaldehydes (class 41) and phenyl diamines (class 56) can be predicted as reactive compounds to both species from the toxic ratios. However, there are some classes that may share different MOAs between the two species. A few classes, such as allylic halogens (class 5) and aldehydes (class 10), are slightly more toxic to fish than to *V*. *fischeri*. By contrast, a number of hydrophilic classes, such as diols (class 7), diones (class 13), phenyl alcohols (class 38), phenones (class 42), benzoic acids (class 45), phenols with alcohol, alkoxy, ketone or ester group (class 49), N-alkyl anilines (class 54), nitrobenzenes (class 59) and pyridines (class 71), exhibit more excess toxicity to *V*. *fischeri* than to fish. Moreover, although some ionizable compounds were identified as baseline or less inert toxicity to both species, more ionizable compounds were identified as reactive chemicals to *V*. *fischeri* than to fish.

## Discussion

### Comparison of toxicities between fish and *V*. *fischeri* for baseline compounds

Analysis of the toxic ratios shows that compounds classified as baselines in fish toxicity can also be identified as baselines in *V*. *fischeri* toxicity. Linear relationship has been observed between log K_OW_ and the toxicities of fish or *V*. *fischeri* for baseline compounds (Fig 2). Very similar slopes and intercepts of regression equations (see Eqs 4 and 5) indicate that the toxicity values to *V*. *fischeri* can be used to estimate the toxicity values to fish for baseline compounds. It also suggests that baseline compounds share the same toxic mechanism and bio-uptake process to both fish and *V*. *fischeri*. This can be explained from the relationship between bioconcentration factor (BCF) and internal critical concentration (or called critical body residue CBR) [12,26]. Where CBR_F_ or BCF_F_ is to fish and CBR_VF_ or BCF_VF_ is to *V*. *fischeri*.

$$BCF=CBR/LC_{50}\;(or\;IBC_{50})$$ (8)

$$\log\;1/LC_{50}\;(or\;\log\;1/IBC_{50})=\log\;1/CBR+\log\;BCF$$ (9)

The log 1/CBR_F_ values vary in a narrow range (CBR = 2–8 mmol/kg in wet weight) with an average of –0.43 (mmol/kg) has previously been reported for neutral hydrophobic baselines in fathead minnow [26,27]. Therefore, if log BCF is linearly related to log K_OW_ for neutral hydrophobic compounds, log 1/LC_50_ (or log 1/IBC_50_) will be linearly related to log K_OW_ as well. A linear relationship between log BCF_F_ and log K_OW_ has been observed for persistent and neutral hydrophobic chemicals (i.e. log K_OW_) in fish by many authors (log BCF = 0.660 log K_OW_− 0.333) [28,29]. This can explain why log 1/LC_50_ is linearly related to log K_OW_ for neutral hydrophobic baseline compounds. On the other hand, the relationship between log BCF_VF_ and log K_OW_ has not been reported in the literature and no determined CBR_VF_ values are available in the literature. The linear log 1/IBC_50_ –log K_OW_ relationship suggests that, like fish bioconcentration, the bioconcentration factor to *V*. *fischeri* (log BCF_VF_) is also linearly related to log K_OW_ for neutral hydrophobic baseline compounds. At the same time, the linear relationships of the toxicities against log K_OW_ with similar slopes and intercepts indicate that the CBRs are close to a constant both to fish and *V*. *fischeri* (i.e. CBR_F_ ≈ CBR_VF_) for neutral hydrophobic baseline compounds. Therefore, it is suggested that the average log 1/CBR_VF_ of neutral hydrophobic baseline compounds (log K_OW_ > 1.5) is close to –0.43 as well. It is noteworthy that CBR values of 2–8 mmol/kg refer to the wet weight for neutral hydrophobic baseline compounds. The whole body wet weight CBR value is believed to be higher for hydrophilic chemicals (i.e. log K_OW_ < 1.5) [30,31]. At the same time, the log BCF is not linearly related to the log K_OW_ for the hydrophilic compounds because K_OW_ itself is no longer the surrogate for BCF and the BCF is predominantly based on water-water partitioning [32].

The linear relationships of the toxicities against log K_OW_ also indicate that compounds used to develop the baseline models (Eqs 4 and 5) should be carefully selected. Although linear relationship between log BCF and log K_OW_ is observed for most of the hydrophobic compounds [27], log BCF is not linearly related with log K_OW_ for all the compounds. For high K_OW_ chemicals (e.g. log K_OW_ > 7), there is partitioning to suspended organic matter in water that lowers the bioavailable (dissolved) fraction of chemicals that is actually absorbed by the organism, therefore, the log BCF were over-estimated by the linear log BCF–log K_OW_ model; At the same time, the log BCF may be over-estimated by the linear log BCF–log K_OW_ model due to their insufficient exposure durations to reach a steady state. It is possible that steady state was not approximated in the time course of the acute toxicity testing and hence data interpretations and comparisons are prone to uncertainty [33,34]. Therefore, the baseline toxicities will not be well calculated if these highly hydrophobic compounds were used in the baseline model development. These compounds should be removed from Eqs 4 and 5.

### Comparison of toxicities between fish and *V*. *fischeri* for less inert compounds

Less inert chemicals do not react with specific receptors in an organism, but are slightly more toxic than baseline toxicity. These chemicals are often characterized with polar functional groups, such as hydroxy and amino, and act by a so-called ‘‘polar narcosis” mechanism [6]. Linear relationships of the toxicities against log K_OW_ with similar slopes and intercepts to both species (Fig 2) indicate that, like baseline compounds, these less inert compounds also share the same toxic mode of action between fish and *V*. *fischeri*.

**Fig 3:**
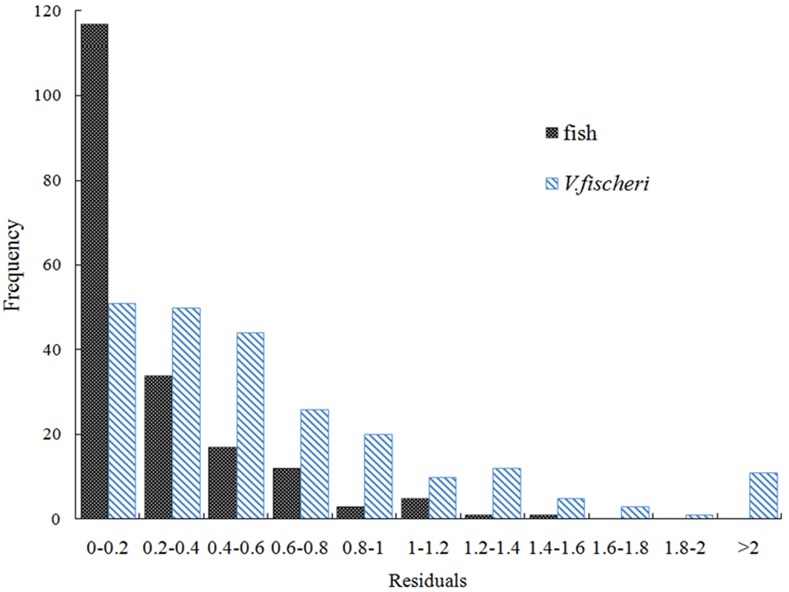
Experimental uncertainty for fish and *V*. *fischeri* toxicities.

Comparison of the toxicities between fish and *V*. *fischeri* for less inert compounds shows that the absolute average residual AAR in *V*. *fischeri* toxicity is greater than that in fish toxicity (AAR = Σ|Determined toxicities–Predicted toxicities from baseline model| / Number of compounds). More compounds with log TR > 1 have been observed in *V*. *fischeri* toxicities than in fish toxicities (see class 2 in Table 1). The same situation was observed for baseline compounds (see class 1 in Table 1). This can be attributed to the greater experimental uncertainty in the toxicity testing to *V*. *fischeri* than to fish. Fig 3 is the histograms of the absolute residuals for 233 compounds to *V*. *fischeri* toxicity and 190 compounds to fish toxicity obtained from different references, respectively. Inspection of the reproducibility of toxicity data contained from different sources shows that the experimental error of *V*. *fischeri* toxicities is quite high with the average absolute residual AAR = 0.64, whereas that of fish toxicity is very low with AAR = 0.22. This also explains why the relationship of the toxicities to *V*. *fischeri* against log K_OW_ is poorer than that to fish for baseline and less inert compounds (see Eqs 4–7).

Although it is well recognized that less inert compounds are slightly more toxic than baseline compounds, no cut-off of toxic ratio has been reported and used to separate less inert compounds from baseline level. The method we used to discriminate baseline and less inert compounds in this paper is based on a comparison of the absolute residuals between the experimental toxicities and the toxicities predicted from baseline or less inert models. If the absolute residual of a compound predicted from baseline model (Eqs 4 or 5) is less than that predicted from less inert model (Eqs 6 or 7), the compound will be predicted as a baseline compound (e.g. As described in detail in the previous paper [11]). Otherwise, it will be predicted as a less inert compound (see Table C in S1 File). The reason for the use of this method is that the equations developed from log K_OW_ appear not to be parallel between baseline and less inert compounds (Fig 2). The log 1/LC_50_ and log 1/IBC_50_ of less inert compounds have increasing shifts towards the baselines with increasing log K_OW_ values. No cut-off can be made from the toxic ratio between baseline and less inert compounds. It is noteworthy that this method can well separate the less inert compounds from baselines for the compounds with log K_OW_ < 4. It is difficult to distinguish the less inert compounds from baseline level for highly hydrophobic compounds. The toxicities of very hydrophobic compounds are well predicted from both baseline and less inert models. For the compounds with log K_OW_ > 4, no significant differences have been observed between the toxicities predicted from baseline and less inert models (Fig 2). The log 1/LC_50_ and log 1/IBC_50_ predicted from less inert models (Eqs 6 and 7) are close to or even less than the values predicted from baseline models (Eqs 4 and 5). That is why some baseline compounds were predicted as less inert compounds and some less inert compounds were predicted as baseline compounds (see N_B_/N_L_ in Table 1). These “so-called” polar compounds with log K_OW_ > 4 can be classified as baseline compounds because the contributions of polar interactions decrease with higher log K_OW_.

### Comparison of toxicities between fish and *V*. *fischeri* for reactive compounds

The reactive compounds may form irreversible covalent bonds with amino acid protein residues or act with specific receptors in a non-covalent manner [4]. They can chemically react with target biomolecules and have various reactive mechanisms with biomolecule and exhibit very different toxicity with greater toxicity than those of baseline and less inert compounds. The log 1/CBR values in Eq 9 are not close to a constant for the compounds. They usually have log 1/CBR values much greater than baseline compounds [35]. Therefore, these compounds exhibit significantly higher toxicity than that predicted from baseline model with log TR > 1. Although the above analysis suggests that non-reactive compounds (i.e. baseline and less inert compounds) share the same MOAs between species, significant differences in the toxic ratios have been observed for some classes of compounds between the two species (Table 1). More compounds were predicted as reactive chemicals to *V*. *fischeri* than to fish, especially for the hydrophilic compounds. The differences in the toxic mechanisms can cause the differences in the toxic ratios between fish and *V*. *fischeri* toxicities. The toxic effect to *V*. *fischeri* is caused by the inhibition of luciferase by a reactive chemical, causing a reduction in the light emission of bioluminescent bacteria [14]. On the other hand, the toxic effect to fish is caused by the reactivity of compounds with nucleophilic sites in peptides, proteins, and nucleic acids. These molecular reactions usually include alkylation of cysteine and amino groups in peptides and proteins as well as the alkylation of nitrogen and oxygen groups in DNA and RNA [36]. The differences in the toxic mechanisms will result in the differences in the CBRs for reactive compounds, leading to the toxic ratios greater than one.

Although some compounds were predicted as reactive compounds to *V*. *fischeri*, but not to fish from the toxic ratios, we still cannot completely ensure whether these compounds share different toxic mechanisms because the toxic ratio calculated from Eqs 1 or 2 is based on the external critical concentration (i.e. LC_50_ or IBC_50_), rather than the internal critical concentration (or called critical body residue, CBR). The toxic ratio is closely related to the exposure route. The real excess toxicity which is used to identify reactive chemicals from baseline level should be based on the internal critical concentration, rather than the external critical concentration. The theoretical relationship of toxic ratio (TR) and internal critical concentration (or CBR) can be derived from the definitions of excess toxicity (Eq 2) and BCF (Eq 8) (see more details in reference [27]).

$$\log\;TR=\log\;1/T_{\;exp}\;\text{-}\;\log\;1/T_{\;pred\;(baseline)}={\left(\log\;1/CBR+\log\;BCF\right)}_{\;\exp}\;\text{-}\;{\left(\log\;1/CBR+\log\;BCF\right)}_{\;pred\;(baseline)}=\Delta \log\;1/CBR+(\log\;BCF_{\;\exp}\;\text{-}\;\log\;BCF_{\;pred\;(baseline)})$$ (10)

The above relationship between log TR and log 1/CBR indicates that there are two possible reasons why some compounds exhibit greater toxic effect to *V*. *fischeri* than to fish. First, if the log BCF of a chemical is well estimated by log K_OW_ (the predicted log BCF is close to the determined log BCF), the log TR will be close to Δlog 1/CBR and the difference in the external critical concentration will reflect the difference in the internal critical concentration (i.e. log TR ≈ Δlog1/CBR). This suggests that the differences in toxic ratios between *V*. *fischeri* and fish species for these compounds will reflect the differences in toxic mechanisms. In other words, these compounds share different toxic mechanisms between species. Second, if the log BCF of a chemical cannot well be estimated, the log TR will not reflect the difference in the internal critical concentration between reactive and baseline level (i.e. log TR ≠ Δlog1/CBR). The under-estimated log BCF value will result in a greater toxic ratio (TR). This suggests although there are differences in the toxic ratios between *V*. *fischeri* and fish species for some compounds (e.g. hydrophilic compounds), they may not have different toxic mechanisms. Inspection of the toxic ratios of the hydrophilic compounds shows that some of them have excess toxicity to both species with log TR > 1, but more compounds were identified as reactive chemicals to *V*. *fischeri* than to fish, with the toxic ratios greater to *V*. *fischeri* than to fish. *V*. *fischeri* are bacterium and fish are aquatic animals. The hydrophilic compounds can more easily pass through the membrane of *V*. *fischeri* than the gill and skin of fish. The great differences in toxic ratios for these hydrophilic compounds may be attributed to the under-estimated log BCF to *V*. *fischeri* as compared to fish. The target site may be located in the aqueous phase in *V*. *fischeri* and log K_OW_ is not an appropriate parameter to describe the bioconcentration potential for these hydrophilic compounds. As discussed above, the log BCF is not linearly related to the log K_OW_ for the hydrophilic compounds because K_OW_ itself is no longer the surrogate for BCF.

If no metabolism occurs, the bioconcentration factors of neutral hydrophobic chemicals obtained at steady state are correlated with hydrophobicity. The hydrophobicity is the principal driving force of bioconcentration for these organic chemicals to fish. Linear relationships have been observed between log BCF and log K_OW_ for many neutral hydrophobic organic compounds in fish bioconcentration [37]. This indicates that log BCF can well be predicted from log K_OW_ for most of neutral hydrophobic organic compounds. The predicted log BCF is close to the experimental log BCF and the log TR reflects the difference in the internal effect concentration. However, it may not be the case for *V*. *fischeri* bioconcentration. *V*. *fischeri* is a lower level of organism and, on the other hand, fish is a high level of organism. To fish, the toxicants need to pass through the gill and skin to have toxic effect. The skin or gill, tissues and organs can restrict the transport of some chemicals, such as hydrophilic compounds, into the target sites, resulting in lower toxicity to fish. Low toxic effects to fish for some hydrophilic compounds do not indicate their low reactivity with the biological macromolecules at the target sites, but due to their low bioconcentration potentials. To *V*. *fischeri*, by contrast, hydrophilic compounds can more easily pass through the cell membrane, react with the target macromolecules and exhibit excess toxicity. The differences in the toxic ratios between the two species may be due to the differences in their physiological structures, leading to the under-estimated log BCF and the greater log TR to *V*. *fischeri* for these hydrophilic compounds. A large prediction error of log BCF from the linear log BCF–log K_OW_ model will result in a wrong prediction of MOA from the log TR.

The equilibrium or steady state is another important factor that can affect the differences of toxic ratios for hydrophilic compounds. The TR values calculated from Eq 10 are based on the condition of equilibrium or steady state. At equilibrium and steady state, the whole body concentrations in the organisms largely reflect the concentrations in the exposure water due to water-water partitioning. *V*. *fischeri* would have a higher relative volume fraction of water than fish because fish have more protein and lipid than *V*. *fischeri*. The hydrophilic compounds may have very significant differences in kinetic processes (such as uptake, elimination or active processes) because of the difference in volume fraction between fish and *V*. *fischeri*, leading to the differences in exposure durations to reach a steady state and then the differences in toxic ratios.

In order to further investigate the relationship of toxic mechanisms between species, the log K_OW_ was added into Eq 3 to improve the interspecies correlation. The hydrophobic parameter log K_OW_ is commonly used to parameterize bio-uptake (or bioconcentration factor) and correct the difference of bio-uptake between species. The resultant equation is:

$$\log\;1/LC_{50}=0.456\;\log\;1/IBC_{50}+0.352\;\log\;K_{OW}+1.39$$ (11)

N = 375 S = 0.79 R^2^ = 0.64 F = 331

Although the interspecies correlation does not increase significantly, the increase of coefficient of determination from 0.55 to 0.64 indicates that the differences in the toxic ratios between fish and *V*. *fischeri* for the hydrophilic compounds can be partly attributed to the differences of bioconcentration. Positive regression coefficient of log K_OW_ in Eq 11 indicates that the increase of hydrophobicity of a chemical will increase the difference between *V*. *fischeri* and fish toxicities. In other words, the greater log K_OW_ value of a chemical, the greater toxicity to fish than that to *V*. *fischeri*. By contrary, the toxicity value of a hydrophilic compound to *V*. *fischeri* can be significantly greater than that to fish because of negative log K_OW_ value in Eq 11.

There are several reasons that can lead to the relatively poor relationship in Eq 11. First, the experimental error is very high for *V*. *fischeri* toxicity based on the analysis of the residuals from the two sources (Fig 3). This can also be seen from the relationships between the toxicities and log K_OW_ to *V*. *fischeri* for baseline and less inert compounds, with coefficients of determination of 0.89 and 0.79 (Eqs 5 and 7), respectively, less than the relationships to fish (Eqs 4 and 6). Second, the bioconcentration cannot be parameterized only by hydrophobicity and the log BCF is not very significantly related to log K_OW_ for all compounds. The coefficients of determination of bioconcentration models based on log K_OW_ vary from 0.59 to 0.94 for a series of diverse compounds [29]. It may explain why the relationship of log 1/LC_50_ with log 1/IBC_50_ and log K_OW_ is not significantly improved.

### Comparison of toxicities between fish and *V*. *fischeri* for outliers

The baseline toxicity is the minimum toxicity that compounds exhibit. The log TR values calculated from Eq 2 should be close to or greater than zero for all the studied compounds. However, 75 compounds in *V*. *fischeri* toxicity and 24 compounds in fish toxicity have been observed with log TR < –1 (see Table 1 and Table C in S1 File). Experimental error in the toxicity test is one of the reasons resulting in these outliers. Greater experimental error to *V*. *fischeri* than to fish indicates a greater number of outliers observed in *V*. *fischeri* toxicity than in fish toxicity. Another important reason can be attributed to the reduced bioavailability (or third phase effect) for the highly hydrophobic compounds [31,34]. The toxicity test exposures exceed water solubility for these compounds, leading to their poor bioavailability and low toxic effects with log TR <–1. Investigation on the bioconcentration in fish shows that the log BCF values were significantly over-estimated for most of compounds with log K_OW_ > 7 (e.g. hexachlorophene, tetrabromobisphenol A, nonane, pentadecanol and PAHs) [27,34]. Although this observation is based on the relationship of log BCF–log K_OW_ in fish, it may be applicable to *V*. *fischeri* because the same characteristics of outliers between fish and *V*. *fischeri* toxicities. Greater over-estimation of log BCF leads to greater differences between determined and calculated log BCF values, and hence to the log TR values calculated from Eq 10 being much less than zero (i.e. log TR < –1).

### Factors that affecting the comparison of toxicities between fish and *V*. *fischeri*

Bioavailability mentioned above is one of the factors that can affect the discrimination of excess toxicity from baseline level. There are several other factors that can affect the difference in toxicities between fish and *V*. *fischeri*. The exposure duration is another factor that can greatly affect toxicities to both *V*. *fischeri* and fish species. 15 min may not be sufficient exposure duration in the microbial test and 96 hours may not be sufficient for fish to approach steady-state/equilibrium. The uptake phase should run for 28 days unless it can be demonstrated that equilibrium has been reached earlier [38]. The uptake times of highly hydrophobic chemicals from water can be even longer [37]. The insufficiency of exposure duration can lead to the acute toxicities being very different from the sub-acute or chronic toxicities for a chemical to the studied species. It can also result in non-linear log BCF–log K_OW_ relationship throughout the entire range of K_OW_ for chemicals in this study. BCF can be significantly over-estimated for highly hydrophobic compounds from the linear log BCF–log K_OW_ equation because of the insufficient exposure duration for 15 min to *V*. *fischeri* and 96 h to fish, leading to the toxicities of highly hydrophobic compounds are significantly lower than the predicted values by baseline models to *V*. *fischeri* and fish, respectively.

Bioconcentration potential can also affect the toxicities to *V*. *fischeri* and fish. First, hydrophilic compounds have greater bioconcentration potential than expected to an organism. The bioconcentration potentials of hydrophilic compounds (e.g. log K_OW_ < 0) are significantly under-estimated from the linear log BCF- log K_OW_ equation [37,39]. At lower K_OW_, the BCF does not continue to decrease with decreasing log K_OW_ because of internal water/external water partitioning. This may in part explain the high TRs for hydrophilic chemicals. Furthermore, fish are higher level of organisms with high lipid content and have greater bioconcentration potential than bacteria for neutral compounds. On the other hand, *V*. *fischeri* are bacteria with high aqueous content and have greater bioconcentration potential than fish for hydrophilic compounds. The BCFs are under-estimated from the linear log BCF–log K_OW_ equation, leading to the log TR calculated from Eq 10 being greater than one. This may explain why more hydrophilic compounds show excess toxicity to *V*. *fischeri* than to fish (Table 1).

Ionization is another factor that can affect the toxicities to organisms. In theory, the log K_OW_ obtained from EPI Suite is for the neutral form. This indicates that the minimum toxicity calculated from the baseline model is for the neutral form. However, some chemicals can be ionized in the toxicity test with more than 50% ionization at the studied pH (Table 1). Investigation on the bioconcentration for ionizable compounds shows that although non-ionized form makes main contribution to BCF, the ionized form also makes partly contribution to BCF [37]. In other words, the apparent/true BCF of an ionizable compound should be less than the predicted BCF developed from neutral compounds. This indicates that the baseline toxicities are over-estimated from the baseline models developed from the neutral compounds for ionizable compounds. If the ionizable compounds were baseline compounds, their toxicities would be less or significantly less than that predicted from the baseline models. Examination of the toxicities in Table 1 shows that only a few ionizable compounds exhibit greatly lower toxicities than baseline toxicity with log TR < –1. Many ionizable compounds show strong toxic effects to the two species with log TR > 1, especially to *V*. *fischeri*. This suggests that most of the ionizable chemicals are reactive compounds. The greater toxic effects to *V*. *fischeri* than to fish are due to the greater bioconcentration potentials to *V*. *fischeri*. *V*. *fischeri* are bacterium and fish are multicellular organism. It has been observed that ionizable compounds have poor bio-uptake in fish by many authors [32]. It may be easier to pass through the cell membrane of bacterium than the gill and skin of fish for the ionizable compounds [37]. It explains why so many ionizable compounds show excess toxicity to *V*. *fischeri* than to fish (Table 1).

## Conclusion

Comparison of the toxicities between *V*. *fischeri* and fish shows that there is chemical-specific species sensitivity between fish and *V*. *fischeri*. Comparison of the log TR for both species indicates most chemical classes can be predicted as the same MOAs between the two species. Baseline or less inert compounds share the same MOAs and bio-uptake process between fish and *V*. *fischeri*. The log BCF is linearly related to log K_OW_ and the CBRs are close to a constant both to fish and *V*. *fischeri* for neutral hydrophobic baseline compounds. Less inert compounds with log K_OW_ > 4 can be identified as baseline compounds because the contributions of polar interactions decrease with higher log K_OW_. However, significant differences in MOAs were also observed between the two species. More compounds were predicted as reactive chemicals to *V*. *fischeri* than to fish especially for hydrophilic and ionizable compounds. These compounds may share different mechanisms between *V*. *fischeri* and fish. However, greater experimental error in *V*. *fischeri* toxicity is also one of the reasons resulting in the differences. Another important reason can be partly due to the under-estimated log BCF of hydrophilic compounds to *V*. *fischeri*. Low toxic effects to fish for some hydrophilic and ionizable compounds are due to their low bioconcentration potentials, rather than their low reactivity with the biological macromolecules. The compounds with high log K_OW_ exhibiting very low toxicity (log TR < –1) to both species indicate that the bioconcentration potential of a chemical plays a very important role in the identification of excess toxicity and MOAs.

## Supporting Information

S1 File Toxicity data to *Vibrio fischeri* and fish. (XLSX)
